# Mevalonate kinase deficiency: an early onset inflammatory bowel disease?

**DOI:** 10.1186/1546-0096-13-S1-O56

**Published:** 2015-09-28

**Authors:** A Martins, D Berrebi, L De Lumley, B Petit, B Pellegrino, A Arion, C Jeanne-Pasquier, T Lequerre, I Pellier, M Guillot, A Faye, O Goullet, B Bader-Meunier, I Melki

**Affiliations:** 1 Hospital Prof. Doutor Fernando Fonseca, E.P.E., Paediatrics, Amadora, Portugal; 2 Hôpital Robert Debré, APHP, Department of Anatomic Pathology, Paris, France; 3 Paediatric Department, CHU Limoges, Limoges, France; 4 CHU Limoges, Department of Anatomic Pathology, Limoges, France; 5 Hôpital d'Enfants Armand-Trousseau, Department of Paediatric Haematology and Oncology, Paris, France; 6 C.H.U. Caen, Paediatric Department, Caen, France; 7 C.H.U. Caen, Department of Anatomic Pathology, Caen, France; 8 C.H.U. Rouen, Rheumatology Department, Rouen, France; 9 C.H.U. d'Angers, Department of Paediatric Immunology and Haematology, Angers, France; 10 Centre Hospitalier Robert Bisson, Paediatric Department, Lisieux, France; 11 Hôpital Robert Debré, APHP, General Paediatric Department, Infectious Diseases and Internal Medicine, Paris, France; 12 Groupe Hospitalier Necker-Enfants Malades, APHP, Department of Paediatric Gastroenterology, Hepatology and Nutrition, Paris, France; 13 Groupe Hospitalier Necker-Enfants Malades, APHP, INSERM U768 and Imagine Foundation, Department of Paediatric Immunology Haematology and Rheumatology, Paris, France

## Introduction

Mevalonate kinase deficiency (MKD) is a rare autoinflammatory, autosomal-recessive defect on MVK gene. Clinical spectrum ranges from recurring febrile attacks to malformations and neurologic disorders. Gastrointestinal symptoms are cardinal. Severe gastrointestinal involvement has been described at the onset.

## Objective

To analyse severe gastrointestinal events (SGE) complicating MKD.

## Patients and methods

Retrospective observational French cohort of MKD patients. SGE were defined as complicated inflammatory involvement, requiring an abdominal surgery and/or enteral/parenteral nutrition. Data were collected from clinical charts provided by the members of the Francophone Society for Paediatric Rheumatism and Inflammatory Diseases (SOFREMIP).

## Results

From a 53-patient cohort, nine presented a SGE (17%). From these, disease onset median age was 1.0 months (0-12); one patient deceased (22 months) from a non-gastrointestinal event. Compound heterozygote mutations were found in 7/8, being Val377Ile the commonest (6/8). The main symptoms during febrile attacks were: diarrhoea (100%, 7/7), lymphadenopathy (89.9%, 8/9), skin lesions, joint pain (85.7%, 6/7 each), aphtous ulcers, abdominal pain (83.3%, 5/6 each), splenomegaly (66.7%, 6/9), hepatomegaly (62.5%, 5/8) and vomiting (57.1%, 4/7). Median mevalonic aciduria: 23.05 mmol/mol of creatinine (P_25_=13.7; P_75_=55.5); median MK activity: 2.2% (P_25_=1.0; P_75_=24.0). The significant co-morbidities found in SGE patients in comparison with the global cohort were: failure-to-thrive in 85.7% (6/7), pulmonary diseases in 37.5% (3/8) and feeding disorders in 28.6% (2/7) (p<0.05).

Severe gastrointestinal involvement was the first event in 6% (3/50), representing 43% (3/7) of patients with severe gastrointestinal disease: abdominal adhesions (66.6%, 6/9) and colitis/enterocolitis (4/9, 44.4%) were mainly found. 87.5% (7/8) needed surgery and 44.4% (4/9) required enteral/parenteral nutrition. Despite digestive resection, disease progression remained; two patients needed re-intervention due to surgical complications. Aphtous/ulcerative damage was the main endoscopic feature (4/9, 44.4%). The most consistent microscopic finding was lymphocytic infiltrates. IL-1 antagonists were the most used/effective treatment (4/9), resulting in with complete remission in all three patients with data available.

## Conclusion

MKD severe gastrointestinal involvement presentation has a non-negligible frequency. It usually appears as an aphtous/ulcerative disease involving any part of the digestive tract or as abdominal adhesions, frequently requiring surgery. The treatment with IL-1 antagonists resulted in complete remission in a majority of treated patients. Thus, MKD should be added to the list of monogenic early-onset inflammatory bowel disease.

